# A specific and rapid method for detecting *Bacillus* and *Acinetobacter* species in Daqu

**DOI:** 10.3389/fbioe.2023.1261563

**Published:** 2023-09-25

**Authors:** Yanwei Wei, Shuyue Zhang, Guikun Guan, Ziran Wan, Ruiming Wang, Piwu Li, Yu Liu, Junqing Wang, Guanhua Jiao, Hao Wang, Chuying Sun

**Affiliations:** ^1^ State Key Laboratory of Biobased Material and Green Papermaking (LBMP), Qilu University of Technology, Jinan, Shandong, China; ^2^ Department of Biological Engineering, Qilu University of Technology, Jinan, Shandong, China; ^3^ Lanling Meijiu Co., Ltd., Lanling, Shandong, China

**Keywords:** specific primer PCR, high-throughput sequencing, Daqu, Baijiu production, *Bacillus*, *Acinetobacter*

## Abstract

Daqu is a spontaneous, solid-state cereal fermentation product used for saccharification and as a starter culture for Chinese Baijiu production. *Bacillus* and *Acinetobacter*, two dominant microbial genera in Daqu, produce enzymes and organic acids that influence the Daqu quality. However, there are no rapid analytical methods for detecting *Bacillus* and *Acinetobacter*. We designed primers specific to the genera *Bacillus* and *Acinetobacter* to perform genetic comparisons using the 16 S rRNA. After amplification of polymerase chain reaction using specific primers, high-throughput sequencing was performed to detect strains of *Bacillus* and *Acinetobacter*. The results showed that the effective amplification rates for *Bacillus* and *Acinetobacter* in Daqu were 86.92% and 79.75%, respectively. Thus, we have devised and assessed a method to accurately identify the species associated with *Bacillus* and *Acinetobacter* in Daqu, which can also hold significance for bacterial typing and identification.

## 1 Introduction

Baijiu is known as an ancient and distinct Chinese distilled spirit worldwide (Tu et al., 2022). It comprises one or more grains, typically sorghum, rice, wheat, barley, or maize. White wine fermentation is a complex process involving a variety of microorganisms in an open environment (Mao et al., 2022). During fermentation, barley is used as a microbial fermenting agent (Zhang et al., 2021) and is an integral part of the fermentation process. In fermentation, Daqu, which is used as a microbial fermenting agent (Zhang et al., 2021), is critical in the formation of Baijiu and is essential to develop the particular aroma associated with Baijiu (Xia et al., 2023). Baijiu and Daqu are highly associated with each other; since ancient times, good quality Daqu has been indispensable for producing good quality Baijiu, and the flavor of Baijiu depends on the quality of Daqu. Various enzymes and fungi are abundant in Daqu (Gao et al., 2022). The quality of Daqu greatly affects its yield and rate of product formation, and *Bacillus* and *Acinetobacter* play important roles. *Bacillus* can hydrolyze proteins and starch (Li et al., 2014), and few *Bacillus* species can metabolize the aromatic components in Baijiu, such as diacetyl (Shibamoto, 2014). *Acinetobacter* is the dominant bacteria in the early stage of liquor brewing and can secrete esterases, lipases (Doolittle and Peterfy, 2010), and pectinases (Hu et al., 2010), in addition to other enzymes. Organic acids (Bangar et al., 2022), fatty acids (Yang et al., 2022), amino acids, higher alcohols (Cordente et al., 2021), oligosaccharides (Rastall, 2010), and other small-molecule precursors are conducive to the production of flavored substances in Baijiu (Xiao et al., 2009).

Microbiological testing is a crucial component of biological and technological research. The most common detection methods for microorganisms in the Baijiu fermentation include agar plate culture counting (Hu et al., 2021) and microscopy methods (Maceda and Terrazas, 2022). These two methods can be used to detect the number of microbial colonies and the individual morphology of Baijiu microorganisms. To overcome the limitations of traditional microbial detection technologies, high-throughput sequencing technologies (Jones, 2010), such as polymerase chain reaction (PCR) (Zhu et al., 2020), real-time fluorescence quantitative PCR (Hancock et al., 2010), flow cytometry (McKinnon, 2018), and other techniques are used to further detect and evaluate the number and size of microorganisms. PCR implemented in this study used a specific primer design resulting in a shorter cycle time, lower cost, and faster detection results than high-throughput sequencing and flow cytometry. Specific primer PCR involves designing the upstream and downstream primers for the target microbial DNA, enabling the analysis of trace amounts of DNA to be substantially amplified. The DNA of microorganisms that was amplified by PCR using specific primers has been reported for *C. perfringens*. Specific downstream primers for *Clostridium perfringens* (Weisburg et al., 1991) were designed for selective amplification of a bacterial strain from the myriad microflora in the sample.

Currently, there are two strategies to determine the presence of *Bacillus* and *Acinetobacter* species in a sample. The first involves plate coating using *Bacillus* or *Acinetobacter* screening media. However, this method is time-consuming and does not enable quantitative analysis. In the second strategy, all microbial communities in the sample can be detected using high-throughput sequencing (Xu et al., 2022), but the protocol is expensive and time-consuming, and the species and genus of *Bacillus* and *Acinetobacter* cannot be accurately determined. Therefore, it is necessary to develop highly specific and rapid analytical methods for *Bacillus* and *Acinetobacter* detection.

In this study, by designing specific primers for amplification and high-throughput sequencing, we obtained information about different species, performed accurate species-level analysis, devised a rapid detection method, and developed tools for designing specific primers for other species.

## 2 Materials and methods

### 2.1 Samples and reagents

**FIGURE 1:**
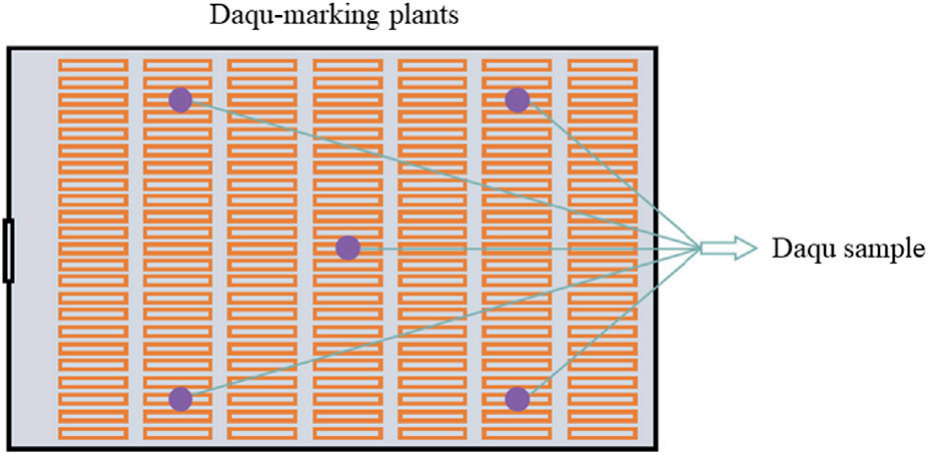
schematic diagram of sampling location for Daqu.

Daqu samples were obtained from mature Daqu at the Shandong Lanling Fine Wine Co., Ltd. production plant. Five Daqu samples from five different points in each room were taken, crushed, mixed as parallel samples (Figure 1), placed in airtight bags, and frozen at −20°C. FastDNA SPIN Kit for Soil and FastPrep were purchased from MP Biomedical lnc. DL2000 Plus DNA Marker, DL 15000 DNA Marker, Phanta Max Super-Fidelity DNA Polymerase and Vazyme Gel Extraction Kit were purchased from Vozymes Biotech Co., Ltd. The sequence alignment software used was Clustalw (https://www.genome.jp/tools-bin/clustalw). Primer synthesis was completed by Sangon Biotech (Shanghai) Co., Ltd.

### 2.2 Method for extracting the Daqu genome

DNA was extracted using the FastDNA SPIN Kit for Soil. First, the solid distillate was lysed, and 500 mg of soil was added to the Lysing Matrix E tube (Lysed DNA) and mixed with the reagents. The mixture was mixed in the FastPrep apparatus for 40 s at a speed setting of 6.0. The supernatant was separated by centrifugation, and 250 μL of PPS was added. The supernatant was separated by centrifugation and the DNA was eluted by adding l mL of the Binding Matrix Supension, while the DNA binding matrix was left in place. Subsequently, we removed the supernatant and transferred 500 μL the mixture to the SPINTM Filter, which was centrifuged and air-dried. Finally, 50 μL of DES was added to elute the DNA (the reagents mentioned in this section are included in the FastDNA SPIN Kit for Soil).

### 2.3 High-throughput macro-genome taxonomic sequencing of Daqu currants

A total of 500 mg of Daqu was placed in a sterilized 2 mL tube, 1× PBS solution was added, and the mixture was shaken and mixed. The sample was centrifuged at 13,000 RCF for 3 min at 25°C, and the top layer was discarded. The 2 mL centrifuge tube was inverted onto blotting paper for 1 min until no liquid was further removed. The first round of PCR amplification was performed after genome extraction using the FastDNA SPIN Kit for Soil Gene Extraction. We used 16 S V3 to V4 zone (Caporaso et al., 2011) universal primers for amplification (338F: 5′-ACT CCT ACG GGA GGC AGC AG-3′ and 806R: 5′-GGA CTA CHV GGG TWT CTA AT-3′). The PCR products were purified and sequenced on the Illumina MiSeq high-throughput sequencing platform.

### 2.4 Primer design and PCR amplification specific to *Bacillus* and *Acinetobacter*

*Bacillus subtilis* 16 S rRNA was used as the template. 16 S rRNA is relatively conserved in structure and function and has changed relatively little during evolution. Although the 16 S rRNA gene sequence is relatively conserved, there are still differences in the 16 S rRNA gene sequence in different microorganisms. In total, 100 16 S rRNA genes of *Bacillus* were searched using NCBI GenBank, and multiple sequence matching was performed using Clustalw to identify gene regions that are highly conserved in the 16 S rRNA of *Bacillus*. Using the identified gene region as a template, Clustalw was used to design primer pairs specific for *Bacillus*. The sequence of this primer pair was: F: 5′-GTC TGT AAC TGA CGC TGA GGC-3′, R: 5′-GCG ATT ACT AGC GAT TCC A-3′. The primers were used to amplify the Daqu DNA.

*Acinetobacter* 16 S rRNA was used as the template. NCBI GenBank was used to search for 100 16 S rRNA genes of *Acinetobacter*, and multiple sequence comparisons were conducted using Clustalw to identify highly conserved gene regions in *Acinetobacter* encoding 16 S rRNA. Using the identified gene region as a template, specific *Acinetobacter* primer pairs were designed using Clustalw. The primer pair sequences used were: F2: (5′-ATG TGA AAT CCC CGA GCT T-3′) and R2: (5′-AGT TTG TCA CTG GCA GTA TCC T-3′). Two specific primers with high homology in *Bacillus* and *Acinetobacter*, but with little homology in non-*Bacillus* and non-*Acinetobacter* with similar sequences, were used for amplification. (Supplementary Figures S1, S2).

The amplification procedure was as follows: pre-denaturation at 94°C for 5 min; 20 cycles of denaturation at 94°C for 1 min, annealing at 55°C for 1 min, and extension at 72°C for 150 s; and extension at 72°C for 10 min using the Phanta Max Super-Fidelity DNA Polymerase (25 µL reactions).

### 2.5 PCR product recovery and sequencing using specific primers

The PCR amplified sample (10 µL) was analyzed on a 2% agarose gel along with the DL 2000 Plus DNA Marker. The Vazyme Gel Extraction Kit was used to recover the PCR amplification products. The *Bacillus* primers used were F: 5′-GTC TGT AAC TGA CGC TGA GGC-3′, R: 5′-GCG ATT ACT AGC GAT TCC A-3′, and *Acinetobacter* primer pairs included F2: 5′-ATG TGA AAT CCC CGA GCT T-3′, R2: 5′-AGT TTG TCA CTG GCA GTA TCC T-3′, which were used to process and analyze Illumina MiSeq high-throughput sequencing data from *Bacillus* and *Acinetobacter*, respectively.

## 3 Results

### 3.1 High-throughput sequencing of bacterial community structure and diversity analysis of bacterial communities in macrophytes

**FIGURE 2:**
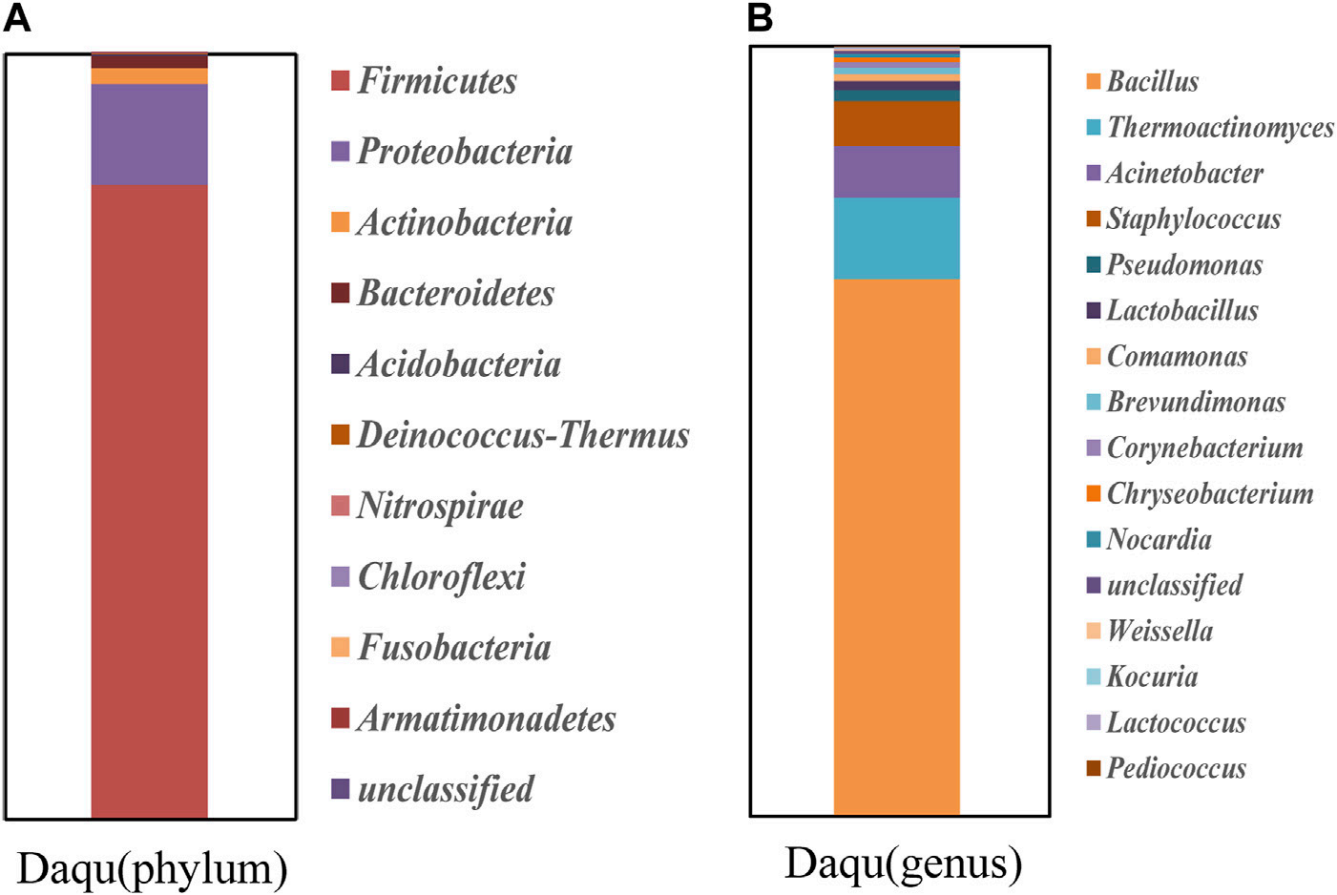
High-throughput sequencing of bacterial community structure and analysis of bacterial community diversity. **(A)** Classification of Daqu samples at phylum level. **(B)** Classification of Daqu samples at the generic level.

The community structure of the Daqu samples at the phylum level was determined based on taxonomic information of the species obtained from the different microbes within the sample (Figure 2A). At the phylum level, a total of 10 community structures relating to bacteria were detected in the two types of Daqu, with a total of four dominant phyla (≥1% relative abundance), namely, *Firmicutes* (82.67%), *Proteobacteria* (13.22%), *Actinobacteria* (7.06%), and *Bacteroidetes* (1.65%). *Firmicutes* were the dominant group of bacteria in the macrophyte samples (Chen et al., 2020).

The community structure of the Daqu samples at the genus level was analyzed from the taxonomic information of the species in the samples (Figure 2B). In the Daqu samples, the top five prokaryotic communities in terms of relative abundance were *Bacillus* (69.61%), *Weissella* (34.33%), *Lactobacillus* (22.07%), *Thermoactinomyces* (19.9%), and *Acinetobacter* (11.56%). *Bacillus* was the dominant genera in the Daqu samples. The protein and starch in Daqu can be decomposed by *Bacillus*, and the aromatic substances in Daqu mainly originate from its action.

### 3.2 PCR analysis of *Bacillus* and *Acinetobacter* from Daqu

**FIGURE 3:**
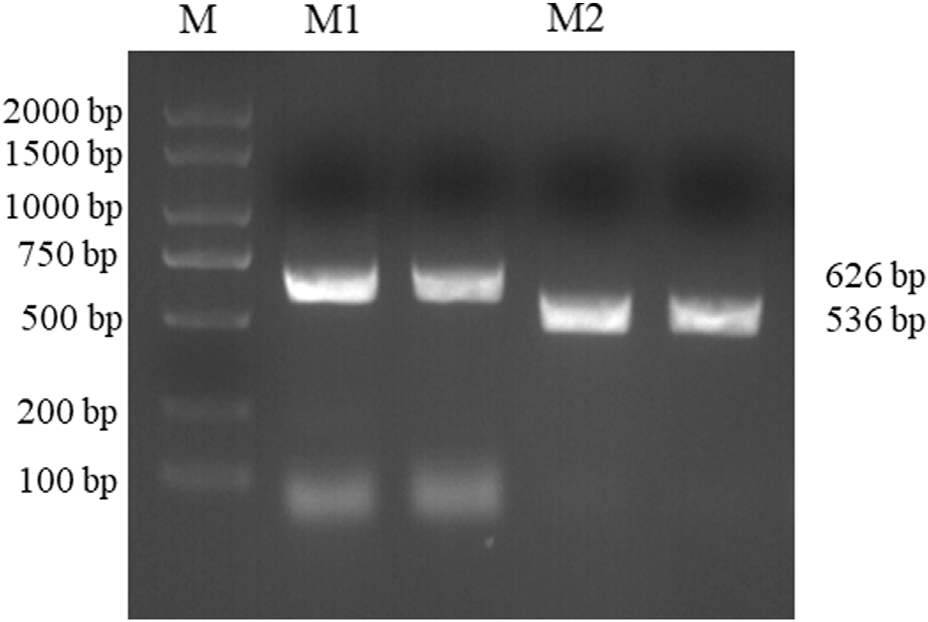
*Bacillus* and *Acinetobacter* agarose gel electrophoresis results. (Lane M: KB Ladder, Lane M1: *Bacillus*, Lane M2: *Acinetobacter*).

DNA was extracted from Daqu samples. The PCR amplification products of *Bacillus* and *Acinetobacter* were 626 and 536 bp, respectively, as shown in Figure 3. Meanwhile PCR experiments at 50, 52, 55, 57 and 60°C were also performed in this paper, and the annealing temperature was chosen at 55°C according to the experimental results (Supplementary Figure S3). The results also showed no spurious bands; thus, the primer pairs for *Bacillus* and *Acinetobacter* were specific.

The amplified bands were purified using a Vazyme Gel Extraction Kit, and the purified products were sent to Sangon Biotech (Shanghai) for sequencing using the Illumina MiSeq PE300 platform and for high-throughput data processing and analysis.

### 3.3 High throughput sequencing for detecting *Bacillus* in Daqu

**FIGURE 4:**
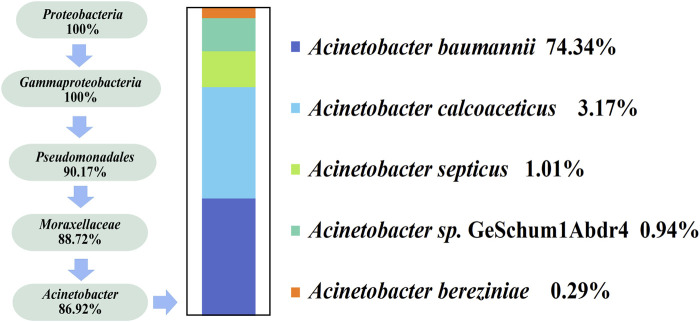
High-throughput sequencing of species classification of *Bacillus*.

The results in Figure 2A show that *Bacillus* mostly contains macromolecules and produces proteases, amylases, and cellulases. We designed specific primers for *Bacillus*, performed specific primer PCR to amplify 16 S rRNA from only one microorganism, and verified the specificity of these primers using high-throughput sequencing (Figure 4). We identified six *Bacillus* species using high-throughput sequencing, and the resulting sequences were uploaded to the NCBI database (PRJNA985236). All six *Bacillus* species, namely, *B. velezensis* (82.07%), *Bacillus paramycoides* (2.89%), *Bacillus licheniformis* (1.6%), *Bacillus sp*. (0.25%), *Bacillus coagulans* (0.086%), and *Bacillus ginsenggisoli* (0.022%), belonged to the phylum *Firmicutes*, with *Bacillus velezensis* being the dominant strain.

### 3.4 High-throughput sequencing for detecting *Acinetobacter* in Daqu

**FIGURE 5:**
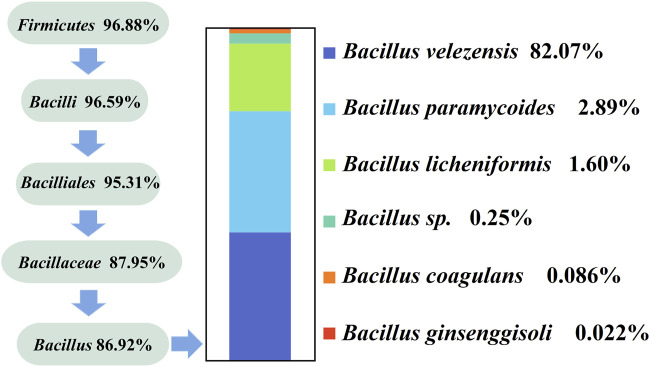
Classification of species of *Acinetobacter* sequenced at high throughput.

As shown in Figure 2B, *Acinetobacter* was the dominant bacterial group in Daqu. We designed specific primers for *Acinetobacter*, performed PCR to selectively amplify the 16 S rRNA, verified the specificity of these primers after amplification, and conducted high-throughput sequencing, as shown in Figure 5. We identified five species of *Acinetobacter* by high-throughput sequencing and the resulting sequences were uploaded to NCBI database (PRJNA985236). These five species were classified as *Proteobacteria* at the phylum level and *Acinetobacter* at the genus level. The five species were *A. baumannii* (74.34%), *Acinetobacter calcoaceticus* (3.17%), *Acinetobacter septicus* (1.01%), *Acinetobacter* sp*.* GeSchum1Abdr4 (0.94%), and *Acinetobacter bereziniae* (0.29%), with *Acinetobacter baumannii* being the dominant strain.

## 4 Discussion

Microbial detection in modern liquor mainly includes high-throughput sequencing technology, PCR, real-time fluorescence quantitative PCR, flow cytometry, and other techniques that can detect the number and biological classification of microorganisms (Table 1). This study used specific primers and high-throughput sequencing analysis to identify Daqu microbial species. At the genus level, two dominant genera (*Bacillus* and *Acinetobacter*) were selected for further high-throughput sequencing. We found that *Bacillus* detected in Daqu included six species: *B. velezensis* (82.07%), *B. paramycoides* (2.89%), *B. licheniformis* (1.6%), *Bacillus* sp. (0.25%), *B. coagulans* (0.086%), and *B. ginsenggisoli* (0.022%). *Bacillus* species are protein hydrolase and amylase producers (Yi et al., 2019) that can enhance the production of organic acids in barley. Additionally, *Bacillus* species can produce protein hydrolases and amylases (He et al., 2019). Of these species, *B. velezensis* (Nam et al., 2009) is an important functional microorganism in winemaking and is the dominant bacterium found in Daqu at high temperature. It has high protease and amylase production capacity and can change the native flora, enzyme activity, and flavor composition of Daqu by regulating the metabolic activity. As an emerging functional strain, it is widely used for food fermentation (Ye et al., 2018). *B. licheniformis* can increase the amount of flavor substances in fermented liquor grains and improve sensory scores (Zhang et al., 2013). High-throughput sequencing showed that the primers F1 (5′-GTC TGT AAC TGA CGC TGA GGC-3′) and R1 (5′-GCG ATT ACT AGC GAT TCC A-3′) were suitable for performing PCR to specifically detect the *Bacillus* species.

**TABLE 1:** Table of main methods and types of microbial detection.

Method	Sample	Detection target	Scope of identification	Source
Plate culture	Fragrance type ditty	*Lactobacillus*	Genus	Hu et al. (2021)
PCR-DGGE	Daqu for sauce-flavor liquor	*Lactobacillus sanfranciscensis*	Genus	Ling et al. (2020)
High throughput sequencing technology	Xiaoqu	*Firmicutes*	Phylum	Wang et al. (2022)
Real-time fluorescence quantitative PCR	Poplar tissue	*Fusarium oxysporum*	Genus	Sa et al. (2021)
Macrotranscriptomics	Siol	*Proteobacteria*	Phylum	Siles et al. (2022)
Flow cytometry	White grain	*Lactobacillus*	Genus	Zhang et al. (2023)
Specific primer PCR	Daqu for Luzhou-flavor liquor	*Bacillus*	Species	This study
Specific primer PCR	Daqu for Luzhou-flavor liquor	*Acinetobacter*	Species	This study

In this study, we identified five species of *Acinetobacter*, namely, *A. baumannii* (74.34%), *A. calcoaceticus* (3.17%), *A. septicus* (1.01%), *Acinetobacter* sp. GeSchum1Abdr4 (0.94%), and *A. bereziniae* (0.29%) via high-throughput sequencing. *Acinetobacter*, the dominant bacterial genus in white wine barley, oxidizes glucose to produce acetic acid, which is one of the main flavor components in Baijiu. *Acinetobacter*, which is widely distributed in nature, is an aerobic, power-negative, gram-negative *Coccobacillus* that has been the focus of several research studies in the medical field (Wong et al., 2017). *Actinobacter* is mostly aerobic and, to a lesser extent, anaerobic, having the capacity to produce antibiotics (Lin and Lan, 2014). *Actinobacter* is also widely present in Daqu, fermented grains, and pit mud and drives the production of metabolites during fermentation. The specific primers F2 (5′-ATG TGA AAT CCC CGA GCT T-3′) and R2 (5′-AGT TTG TCA CTG GCA GTA TCC T-3′) for *Acinetobacter* are useful for high-throughput second-generation sequencing analysis. This method can be used to detect more types of *Acinetobacter* and better understand their role as dominant strains in Daqu.

Weisburg et al. (1991) amplified *Clostridium* 16 S rRNA using specific primers and determined the genus specificity of the bacteria, enabling distinction between strains. By ensuring genus specificity, data can be improved, especially for 16 S rRNA-based typing of bacteria (Sanschagrin and Yergeau, 2014). Liu et al. (2022) screened the 16 S rDNA fragments of *Lactobacillus* obtained using *Lactobacillus*-specific primer PCR with a reporter system to assess the activation of natural target sequences by different crRNAs and detected the production of fluorescent signals. In the study, two specific primer pairs were designed to target the highly conserved region of the prokaryotic gene 16 S rRNA: *Bacillus* F (5′-GTC TGT AAC TGA CGC TGA GGC-3′), R (5′-GCG ATT ACT AGC GAT TCC A-3′) and *Actinobacter* F2 (5′-ATG TGA AAT CCC CGA GCT T-3′), R2 (5′-AGT TTG TCA CTG GCA GTA TCC T-3′). The results showed that the prevalence of *Bacillus* and *Acinetobacter* in Daqu was 86.92% and 79.75%, respectively.

In summary, we performed PCR using specific primers to identify the species of various microorganisms and obtain nucleotide sequences of the bacteria, demonstrating an important method for bacterial typing. Compared with traditional 16 S rRNA taxonomic sequencing and high-throughput sequencing with specific primers, the method used in this study can reveal more phylum and genus classes. Therefore, for specific microbial genera such as *Bacillus*, additional information for different species can be obtained, along with a more accurate analysis of the species level. Our present study demonstrates that this method can be used as a rapid detection method when designing specific primers for other species. Relative to high-cost flow cytometry and long-cycle bacterial sequencing, specific primer PCR can amplify the required strains in a short time at a low cost and achieve 79.75% accuracy, making it a widely applicable method.

## Data Availability

The datasets presented in this study can be found in online repositories. The names of the repository/repositories and accession number(s) can be found below: https://www.ncbi.nlm.nih.gov/genbank/, PRJNA985236.

## References

[B1] Bangar S. P. Suri S. Trif M. Ozogul F. (2022). Organic acids production from lactic acid bacteria: a preservation approach. Food Biosci. 46, 2212–4249. 10.1016/j.fbio.2022.101615

[B2] Caporaso J. G. Lauber C. L. Walters W. A. Berg-Lyons D. Lozupone C. A. Turnbaugh P. J. (2011). Global patterns of 16S rRNA diversity at a depth of millions of sequences per sample. Proc. Natl. Acad. Sci. U. S. A. 108, 4516–4522. 10.1073/pnas.1000080107 20534432 PMC3063599

[B3] Chen L. Li Y. Z. Jin L. He L. Ao X. L. Liu S. L. (2020). Analyzing bacterial community in pit mud of Yibin Baijiu in China using high throughput sequencing. Peer J. 8 (1), e9122. 10.7717/peerj.9122 32435541 PMC7227652

[B4] Cordente A. G. Nandorfy D. E. Solomon M. Schulkin A. Kolouchova R. Francis I. L. (2021). Aromatic higher alcohols in wine: implication on aroma and palate attributes during chardonnay aging. Molecules 26 (16), 4979. 10.3390/molecules26164979 34443564 PMC8400268

[B5] Doolittle M. H. Peterfy M. (2010). Mechanisms of lipase maturation. Clin. Lipidol. 5 (1), 117–130. 10.2217/clp.09.84 PMC2883275 20543905

[B6] Gao L. Zhou J. He G. (2022). Effect of microbial interaction on flavor quality in Chinese baijiu fermentation. Front. Nutr. 9, 960712. 10.3389/fnut.2022.960712 35990321 PMC9381987

[B7] Hancock D. Funnell A. Jack B. Johnston J. (2010). Introducing undergraduate students to real-time PCR. Biochem. Mol. Biol. Educ. 38 (5), 309–316. 10.1002/bmb.20414 21567850

[B8] He G. Q. Dong Y. Huang J. Wang X. J. Zhang S. Y. Wu C. D. (2019). Alteration of microbial community for improving flavor character of Daqu by inoculation with *Bacillus velezensis* and *Bacillus subtilis* . Lwt-Food Sci. Technol. 111, 1–8. 10.1016/j.lwt.2019.04.098

[B39] Hu T. L. (2010). Study on enzymatic properties of pectinase. 12th International Wool Research Conference (IWRC 2010), Shanghai, China.

[B9] Hu Y. L. Wang L. Y. Zhang Z. J. Yang Q. Chen S. X. Zhang L. (2021). Microbial community changes during the mechanized production of light aroma Xiaoqu Baijiu. Biotechnol. Biotechnol. Equip. 35 (1), 487–495. 10.1080/13102818.2021.1892525

[B11] Jones W. J. (2010). High-throughput sequencing and metagenomics. Estuaries Coasts 33 (4), 944–952. 10.1007/s12237-009-9182-8

[B12] Li Z. Bai Z. Wang D. Zhang W. Zhang M. Lin F. (2014). Cultivable bacterial diversity and amylase production in three typical D aqus of C hinese spirits. Int. J. Food Sci. Technol. 49 (3), 776–786. 10.1111/ijfs.12365

[B13] Lin M. F. Lan C. Y. (2014). Antimicrobial resistance in *Acinetobacter baumannii*: from bench to bedside. World J. Clin. Cases 2 (12), 787–814. 10.12998/wjcc.v2.i12.787 25516853 PMC4266826

[B14] Ling Y. X. Li W. Y. Tong T. Li Z. M. Li Q. Bai Z. H. (2020). Assessing the microbial communities in four different Daqus by using PCR-DGGE, PLFA, and biolog analyses. Pol. J. Microbiol. 69 (1), 27–37. 10.33073/pjm-2020-004 PMC7256838 32067441

[B15] Liu Y. F. Wang M. C. Yang F. Zhang X. L. Li J. H. Du G. C. (2022). A CRISPR-cas12a-based assay for efficient quantification of *Lactobacillus* panis in Chinese Baijiu brewing microbiome. Fermentation-Basel 8 (2), 88. 10.3390/fermentation8020088

[B16] Maceda A. Terrazas T. (2022). Fluorescence microscopy methods for the analysis and characterization of lignin. Polym. -Basel 14 (5), 961. 10.3390/polym14050961 PMC8912355 35267784

[B17] Mao F. Huang J. Zhou R. Qin H. Zhang S. Cai X. (2022). Effects of different Daqu on microbial community domestication and metabolites in Nongxiang Baijiu brewing microecosystem. Front. Microbiol. 13, 939904–904. 10.3389/fmicb.2022.939904 35847071 PMC9279870

[B18] McKinnon K. M. (2018). Flow cytometry: an overview. Curr. Opin. Immunol. 120, 5.1.1–5. 10.1002/cpim.40 PMC5939936 29512141

[B19] Nam M. H. Park M. S. Kim H. G. Yoo S. J. (2009). Biological control of strawberry *Fusarium wilt* caused by *Fusarium oxysporum f.* sp. fragariae using *Bacillus velezensis* BS87 and RK1 formulation. J. Microbiol. Biotechnol. 19 (5), 520–524. 10.4014/jmb.0805.333 19494701

[B20] Rastall R. A. (2010). Functional oligosaccharides: application and manufacture. Annu. Rev. Food Sci. Technol. 1, 305–339. 10.1146/annurev.food.080708.100746 22129339

[B21] Sa R. B. Zhang J. L. Sun J. Z. Gao Y. X. (2021). Colonization characteristics of poplar fungal disease biocontrol bacteria N6-34 and the inhibitory effect on pathogenic fungi by real-time fluorescence quantitative PCR detection. Curr. Microbiol. 78 (8), 2916–2925. 10.1007/s00284-021-02529-2 34047833

[B22] Sanschagrin S. Yergeau E. (2014). Next-generation sequencing of 16S ribosomal RNA gene amplicons. J. Vis. Exp. (90), 51709. 10.3791/51709 25226019 PMC4828026

[B23] Shibamoto T. (2014). Diacetyl: occurrence, analysis, and toxicity. J. Agric. Food Chem. 62 (18), 4048–4053. 10.1021/jf500615u 24738917

[B24] Siles J. A. Starke R. Martinovic T. Fernandes M. L. P. Orgiazzi A. Bastida F. (2022). Distribution of phosphorus cycling genes across land uses and microbial taxonomic groups based on metagenome and genome mining. Soil Biol. Biochem. 174, 108826. 10.1016/j.soilbio.2022.108826

[B25] Tu W. Cao X. Cheng J. Li L. Zhang T. Wu Q. (2022). Chinese baijiu: the perfect works of microorganisms. Front. Microbiol. 13, 919044. 10.3389/fmicb.2022.919044 35783408 PMC9245514

[B26] Wang Q. Wang C. Y. Xiang X. Q. Xu H. L. Han G. Q. (2022). Analysis of microbial diversity and succession during Xiaoqu Baijiu fermentation using high-throughput sequencing technology. Eng. Life Sci. 22 (7), 495–504. 10.1002/elsc.202200015 35865650 PMC9288988

[B27] Weisburg W. G. Barns S. M. Pelletier D. A. Lane D. J. (1991). 16S ribosomal DNA amplification for phylogenetic study. J. Bacteriol. 173 (2), 697–703. 10.1128/jb.173.2.697-703.1991 1987160 PMC207061

[B28] Wong D. Nielsen T. B. Bonomo R. A. Pantapalangkoor P. Luna B. Spellberg B. (2017). Clinical and pathophysiological overview of *acinetobacter* infections: a century of challenges. Clin. Microbiol. Rev. 30 (1), 409–447. 10.1128/cmr.00058-16 27974412 PMC5217799

[B29] Xia Y. Luo H. Wu Z. Zhang W. (2023). Microbial diversity in jiuqu and its fermentation features: saccharification, alcohol fermentation and flavors generation. Appl. Microbiol. Biotechnol. 107 (1), 25–41. 10.1007/s00253-022-12291-5 36472652

[B30] Xiao Z. Ma C. Xu P. Lu J. R. (2009). Acetoin catabolism and acetylbutanediol formation by *Bacillus pumilus* in a chemically defined medium. Public Libr. Sci. One 4 (5), e5627. 10.1371/journal.pone.0005627 PMC2680964 19461961

[B31] Xu B. Y. Xu S. S. Cai J. Sun W. Mu D. D. Wu X. F. (2022). Analysis of the microbial community and the metabolic profile in medium-temperature Daqu after inoculation with *Bacillus licheniformis* and *Bacillus velezensis* . Lwt-Food Sci. Technol. 160, 113214. 10.1016/j.lwt.2022.113214

[B32] Yang K. Yu M. L. Zhu X. L. Xia Y. Li F. H. Li Y. Z. (2022). Genetic Incorporation of fluorescent amino acid into fatty acid binding protein for fatty acid detection. J. Mol. Biol. 434 (8), 167498. 10.1016/j.jmb.2022.167498 35183558

[B33] Ye M. Tang X. Yang R. Zhang H. Li F. Tao F. (2018). Characteristics and application of a novel species of *Bacillus: bacillus velezensis* . ACS Chem. Biol. 13 (3), 500–505. 10.1021/acschembio.7b00874 29309732

[B34] Yi Z. L. Jin Y. L. Xiao Y. Chen L. C. Tan L. Du A. P. (2019). Unraveling the contribution of high temperature stage to Jiang-flavor Daqu, a liquor starter for production of Chinese Jiang-flavor Baijiu, with special reference to metatranscriptomics. Front. Microbiol. 10, 472. 10.3389/fmicb.2019.00472 30930875 PMC6423406

[B35] Zhang Q. Chen X. Ding Y. Ke Z. Zhou X. Zhang J. (2021). Diversity and succession of the microbial community and its correlation with lipid oxidation in dry-cured black carp (*Mylopharyngodon piceus*) during storage. Food Microbiol. 98, 103686. 10.1016/j.fm.2020.103686 33875196

[B36] Zhang R. Wu Q. Xu Y. (2013). Aroma characteristics of Moutai-flavour liquor produced with *Bacillus licheniformis* by solid-state fermentation. Lett. Appl. Microbiol. 57 (1), 11–18. 10.1111/lam.12087 23594087

[B37] Zhang Z. Y. Wei Y. W. Peng Z. H. Du P. Du X. Y. Zuo G. Y. (2023). Exploration of microbiome diversity of stacked fermented grains by flow cytometry and cell sorting. Front. Microbiol. 14, 1160552. 10.3389/fmicb.2023.1160552 37051523 PMC10083240

[B38] Zhu H. Zhang H. Xu Y. Laššáková S. Korabečná M. Neužil P. (2020). PCR past, present and future. Biotechniques 69 (4), 317–325. 10.2144/btn-2020-0057 32815744 PMC7439763

