# Supplementary material for: A specific and rapid method for detecting Bacillus and Acinetobacter species in Daqu
Source: Front Bioeng Biotechnol. 2023 Sep 25;11:1261563. doi: 10.3389/fbioe.2023.1261563 (PMC10561003; doi:10.3389/fbioe.2023.1261563)
Supplement: Supplementary file 1 [file DataSheet1.docx]

***Supplementary Material***


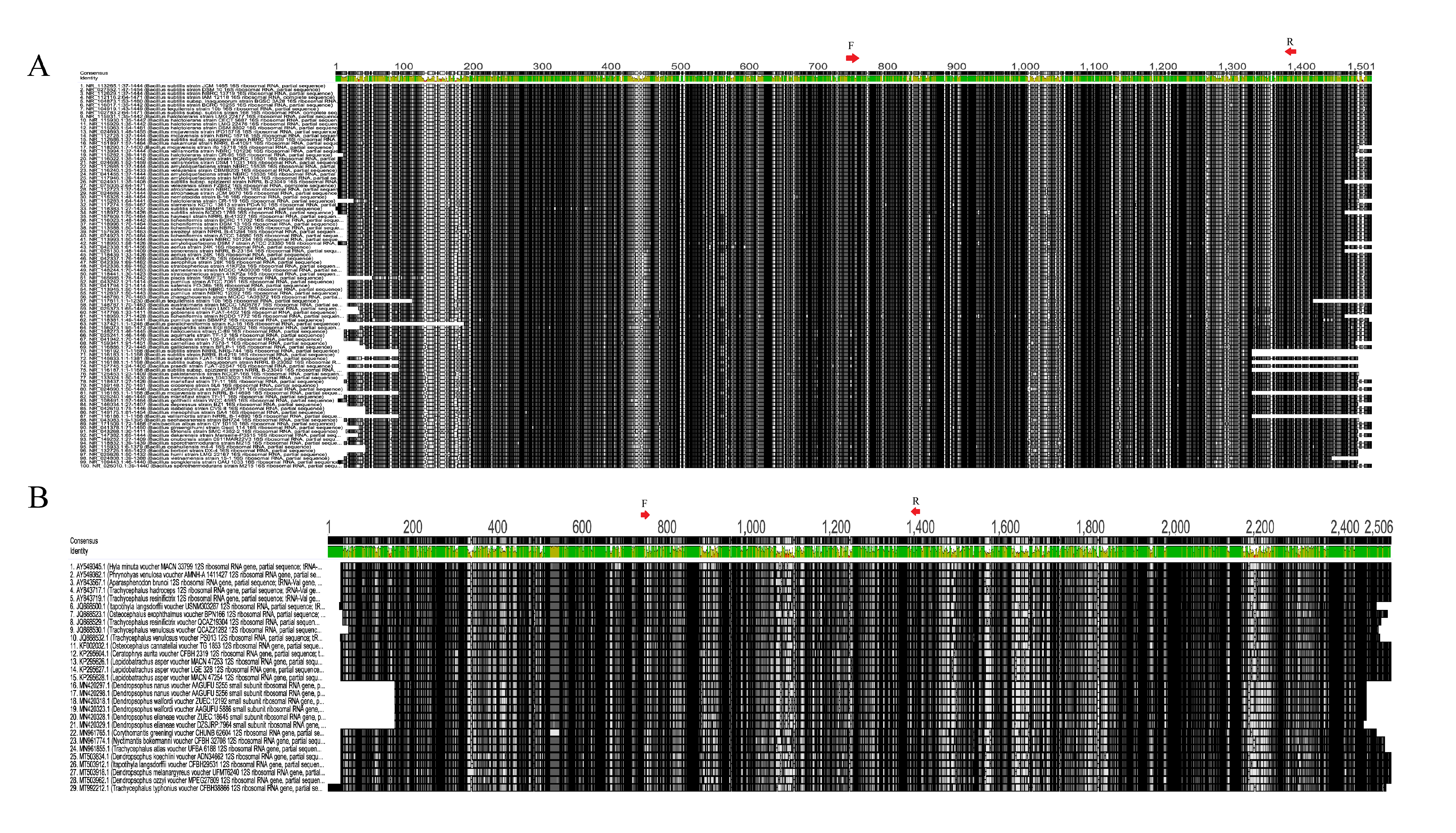


**Fig. S1.** **Gene sequence comparison of *Bacillus*. (A): The specific primers were in the homologous region of *Bacillus*; (B): The specific primers were in the homologous region of non- *Bacillus*.**

**
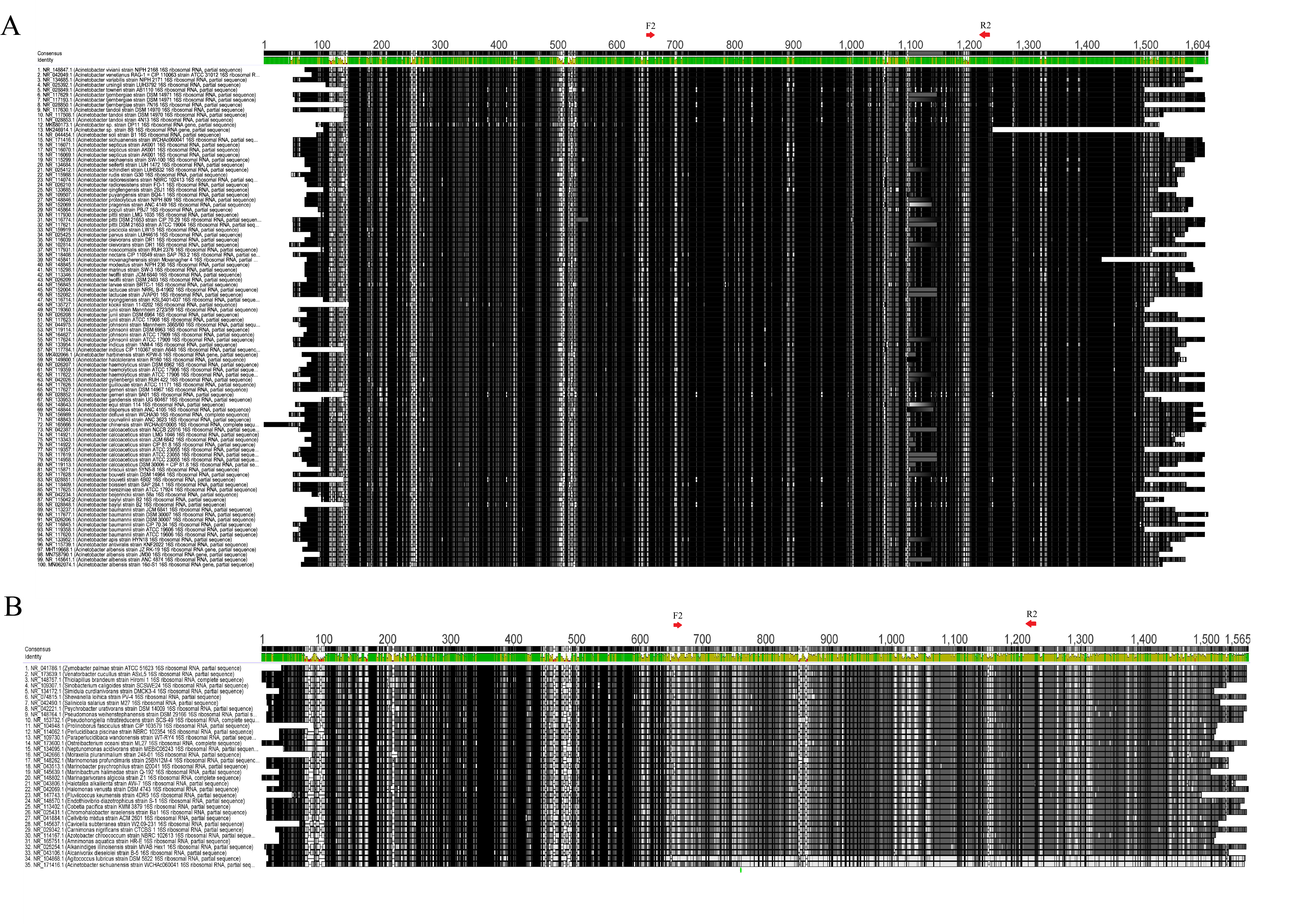
**

**Fig. S2. Gene sequence comparison of *Acinetobacter*. (A): The specific primers were in the homologous region of *Acinetobacter*; (B): The specific primers were in the homologous region of non-*Acinetobacter*.**

**
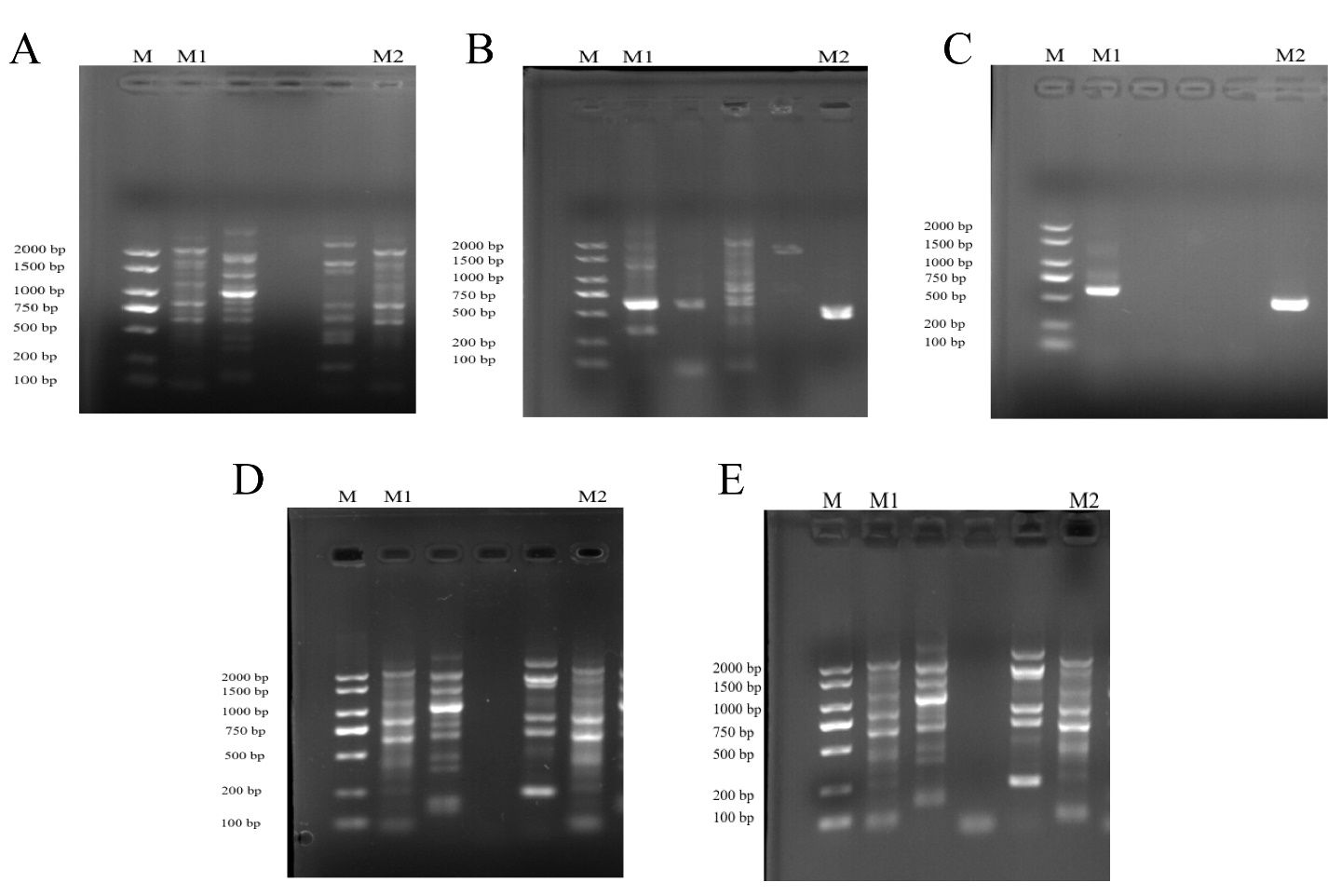
**

**Fig. S3. DNA nucleic acid electrophoresis. (Lane M: KB Ladder, Lane M1: *Bacillus*, Lane M2: *Acinetobacter*) (A): DNA nucleic acid electrophoresis at annealing temperature of 50 °C. (B): DNA nucleic acid electrophoresis at annealing temperature of 52 °C. (C): DNA nucleic acid electrophoresis at annealing temperature of 55 °C. (D): DNA nucleic acid electrophoresis at annealing temperature of 57 °C. (E): DNA nucleic acid electrophoresis at annealing temperature of 60 °C.**
